# An inducible transgenic mouse breast cancer model for the analysis of tumor antigen specific CD8^+^ T-cell responses

**DOI:** 10.18632/oncotarget.5750

**Published:** 2015-10-19

**Authors:** Michael Bruns, Jara Wanger, Olaf Utermöhlen, Wolfgang Deppert

**Affiliations:** ^1^ Heinrich-Pette-Institute, Leibniz-Institute for Experimental Virology, Hamburg, Germany; ^2^ Institute for Medical Microbiology, Immunology and Hygiene, Medical Center and Center for Molecular Medicine Cologne (CMMC), University of Cologne, Cologne, Germany; ^3^ Institute for Tumor Biology, University Medical Center Hamburg-Eppendorf (UKE), University of Hamburg, Hamburg, Germany; ^4^ Present Address: Woldsenweg 7, 20249, Hamburg, Germany

**Keywords:** transgenic breast cancer mouse model, SV40 T-antigen, LCMV NP-epitope, CTL response, differential immune reactivity

## Abstract

In Simian virus 40 (SV40) transgenic BALB/c WAP-T mice tumor development and progression is driven by SV40 tumor antigens encoded by inducible transgenes. WAP-T mice constitute a well characterized mouse model for breast cancer with strong similarities to the corresponding human disease. BALB/c mice mount only a weak cellular immune response against SV40 T-antigen (T-Ag). For studying tumor antigen specific CD8^+^ T-cell responses against transgene expressing cells, we created WAP-T_NP_ mice, in which the transgene additionally codes for the NP_118–126_-epitope contained within the nucleoprotein of lymphocytic choriomeningitis virus (LCMV), the immune-dominant T-cell epitope in BALB/c mice. We then investigated in WAP-T_NP_ mice the immune responses against SV40 tumor antigens and the NP-epitope within the chimeric T-Ag/NP protein (T-Ag_NP_). Analysis of the immune-reactivity against T-Ag in WAP-T and of T-Ag_NP_ in WAP-T_NP_ mice revealed that, in contrast to wild type (wt) BALB/c mice, WAP-T and WAP-T_NP_ mice were non-reactive against T-Ag. However, like wtBALB/c mice, WAP-T as well as WAP-T_NP_ mice were highly reactive against the immune-dominant LCMV NP-epitope, thereby allowing the analysis of NP-epitope specific cellular immune responses in WAP-T_NP_ mice. LCMV infection of WAP-T_NP_ mice induced a strong, LCMV NP-epitope specific CD8^+^ T-cell response, which was able to specifically eliminate T-Ag_NP_ expressing mammary epithelial cells both prior to tumor formation (i.e. in cells of lactating mammary glands), as well as in invasive tumors. Elimination of tumor cells, however, was only transient, even after repeated LCMV infections. Further studies showed that already non-infected WAP-T_NP_ tumor mice contained LCMV NP-epitope specific CD8^+^ T-cells, albeit with strongly reduced, though measurable activity. Functional impairment of these ‘endogenous’ NP-epitope specific T-cells seems to be caused by expression of the programmed death-1 protein (PD1), as anti-PD1 treatment of splenocytes from WAP-T_NP_ tumor mice restored their activity. These characteristics are similar to those found in many tumor patients and render WAP-T_NP_ mice a suitable model for analyzing parameters to overcome the blockade of immune checkpoints in tumor patients.

## INTRODUCTION

Breast cancer is the leading cause of cancer deaths among women in industrialized countries and despite improved diagnostic and therapeutic options accounts for 23% of the total cancer cases and 14% of cancer deaths [1]. In order to achieve a reduction in breast cancer mortality, it thus is vital to improve breast cancer treatment, specifically treatments inhibiting metastatic spread of disseminated tumor cells.

In addition to conventional treatments after surgical removal of the primary tumor, like chemo- and radio-therapy, immune-therapy might develop as a promising option. It thus is vital to understand the mechanisms of anti-tumor immune responses for a given tumor entity and the ensuing mechanisms of immune evasion.

For a detailed study of mammary carcinogenesis, and of immune reactions during early and late processes of tumor development and progression, we used the BALB/c mouse based WAP-T model, a well characterized immune-competent mouse model for oncogene-induced mammary carcinogenesis. Upon induction of the whey acidic protein (WAP) promoter by lactotrophic hormones via mating, expression of SV40 T-antigens (T-Ag, small t, and 17kT antigens) drives transformation of mammary epithelial cells and ultimately tumor growth [2, 3]. Additional expression of mutant p53 in bi-transgenic WAP-T/WAP-mutp53 bi-transgenic mice aggravates tumor progression, and enhances metastasis to the lungs [3, 4]. The clinical relevance of the WAP-T mouse model is emphasized by comparison with human ductal carcinoma *in situ* [3, 5] and molecular similarities between invasive WAP-T and human triple-negative mammary carcinoma subtypes [6, 7]. These carcinomas represent about 20% of all ductal mammary carcinomas and are characterized by bad prognosis.

**Figure 1:**
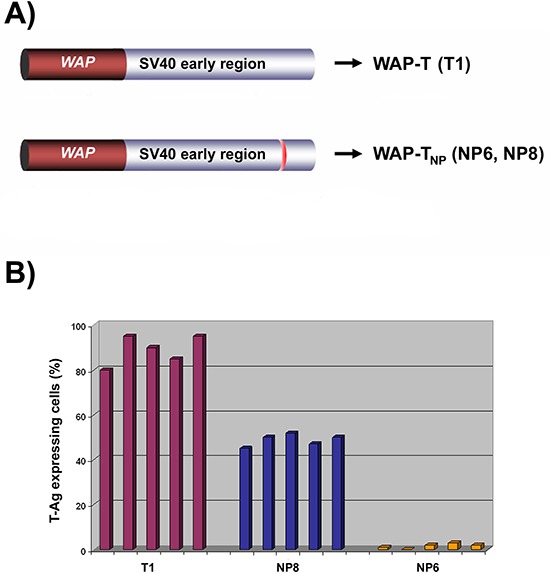
Transgenic mouse lines WAP-T and WAP-T_NP_ **A.** Transgene arrangements. In BALB/c WAP-T mice, the SV40 early gene region under control of the WAP promoter codes for the SV40 early proteins T-Ag, small t, and 17kT, e.g. in the T1 mice used in this study (above). WAP-T_NP_ mice, in addition, code for the strong MHC class I H2^d^ restricted LCMV T-cell epitope NP_118–126_, inserted as a 33 bp oligonucleotide into a transformation-irrelevant carboxy-terminal region of T-Ag (for details see Schulze-Garg et al. [5]); NP6 and NP8 mice were selected for further studies (see Materials and methods). **B.** Distribution of T-Ag expressing cells in lactating mammary glands of T1, NP8, and NP6 mice (immune histology) The percentage of T-Ag positive cells detected in lactating mammary glands (7 days pp) of five individual T1, NP8, and NP6 mice each was evaluated.

H-2^d^-restricted BALB/c mice are considered as “low responders” in terms of a specific CD8^+^ cytotoxic T lymphocyte (CTL) response towards SV40 T-Ag [8]. Nevertheless, protective cellular immunity against transplantable murine SV40 tumors can be achieved by pre-immunization with SV40 or purified T-Ag, which induces an efficient and long-lasting CD4^+^ helper T-cell dependent CTL response against established SV40 tumor cells (e.g. mKSA) [9, 10]. As the T-Ag specific CTL response in BALB/c mice is weak, and as, furthermore, the major histocompatibility complex (MHC) class I H-2^d^ restricted T-Ag specific T-cell epitopes have not yet been characterized, the analysis of T-Ag specific CD8^+^ T-cell responses in BALB/c mice is technically difficult. To allow the epitope-specific analysis of a well-defined CD8^+^ T-cell response against a tumor antigen in WAP-T mice, we inserted the coding sequence (a 33 bp oligomer) for the MHC class I H-2^d^-restricted T-cell epitope NP_118–126_ of LCMV into a transformation-irrelevant C-terminal region of T-Ag, to obtain WAP-T_NP_ mice (Fig. 1A, a detailed description of the WAP-T/WAP-T_NP_ mice used in this study is given in Materials and Methods.) [2]. The H-2^d^-restricted LCMV NP-epitope is dominant in BALB/c mice, as recognition of this motif by specific CTLs leads to virus clearance within 14 days after infection [11]. We previously had shown that immunization of mice with chimeric recombinant T-Ag proteins carrying this epitope induces a strong CTL response [12]. Expression of the chimeric *T-Ag-NP* gene thus should allow the NP-epitope specific analysis of the CD8^+^ T-cell immune response against the T-Ag_NP_ tumor antigen after LCMV infection, if WAP-T_NP_ mice are able to mount a cellular immune response against this epitope. As the immune reactions in LCMV infected BALB/c mice are very well characterized [13], comparative analyses of LCMV infected BALB/c and of WAP-T_NP_ tumor mice should provide additional tools for the characterization of NP-epitope specific immune reactions in WAP-T_NP_ mice at different stages of tumor development and progression. Likewise, comparison of immune reactions in WAP-T_NP_ mice, presenting the NP-epitope, and in WAP-T mice, not presenting the NP-epitope, further enhance the NP-epitope specificity of the WAP-T_NP_ model for the analysis of an NP-epitope specific CTL response.

We here report that in contrast to wtBALB/c mice, WAP-T and WAP-T_NP_ mice are immunologically non-reactive against SV40 T-Ag, but, like wtBALB/c mice, are highly reactive against LCMV. Infection of WAP-T_NP_ mice with LCMV leads to elimination of T-Ag_NP_ expressing cells, both prior to tumor formation as well as in invasive tumors. As the immune-dominant LCMV NP-epitope in T-Ag_NP_ is the only LCMV derived T-cell epitope in NP8 mice, elimination of T-Ag_NP_ expressing cells is NP-epitope specific. Elimination of tumor cells, however, is only transient. Interestingly, even without LCMV infection, tumor-bearing WAP-T_NP_ mice contain LCMV NP-epitope specific CD8^+^ T-cells with low, but measurable CTL activity. CTL activity of these T-cells could be at least partially restored by anti-PD1 treatment, indicating that the endogenous cellular anti-tumor response gets compromised by expression of PD1 and/or PD-L1.

## RESULTS

### NP8 mice are non-reactive against SV40 T-Ag, but highly reactive against the immune-dominant LCMV NP-epitope

**Figure 2:**
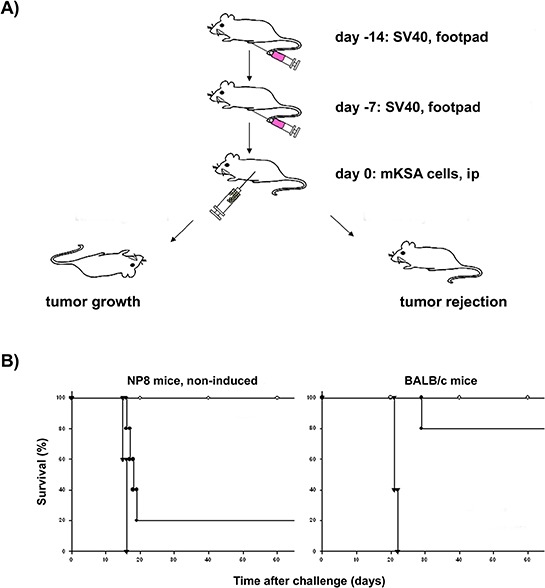
Impaired immune reaction against SV40 T-Ag in NP8 mice **A.** Immunization protocol for SV40 (red syringe) and mKSA tumor cells (clear syringe) in the “mKSA tolerance test“. Mice mounting an immune response against T-Ag are protected against transplanted mKSA cells (“tumor rejection”), while mice with an impaired immune response die from tumor growth. **B.** In contrast to BALB/c mice, naïve (e.g. non-induced) NP8 mice succumb to tumor growth as a consequence of a reduced immune reaction against T-Ag presenting mKSA cells. Five mice per group (*n* = 5) were either infected with 10^5^ PFU VV-941T only (○), treated with 10^5^ mKSA cells only (▼), or a combination of 10^5^ PFU of VV-941T and 10^5^ mKSA cells was applied (●) total observation time was 60 days.

Transgenic expression of T-Ag in T1 or of T-Ag_NP_ in NP8 mice (see Fig. 1) could have various immunologic outcomes, e.g. induction of antigen-specific tolerance, ignorance of the antigen, or activation of a specific immune response. First, the immune-reactivity of T1 and NP8 mice against T-Ag was characterized by immunizing mice with infectious SV40 and compared to that of wtBALB/c mice, followed by challenge with mKSA SV40 tumor cells (see scheme in Fig. 2A).

After injection of 10^6^ mKSA cells, i.e. the ten-thousand-fold dose of tumor cells lethal for 50% of the mice [10], all naïve wtBALB/c mice (13/13 mice) succumbed to mKSA tumors within a mean survival time of 21 days (Table I). Immunization by two sc injections of SV40 established long-term protection against mKSA tumors in almost all (12/13 mice) wt mice.

**Table 1:** Despite prior immunization with SV40, WAP-T and WAP-T_NP_ mice succumb to challenge with SV40 T-Ag expressing mKSA tumor cells as rapidly as naïve wt mice

Mice	Tumor incidence^a^	Mean survival time (days)
wt, naïve	13/13	21
wt, T-Ag immunized	1/13	>100
T1, T-Ag immunized	6/6	18
NP8, T-Ag immunized	11/11	20

a T1 or NP8 mice or wtBALB/c mice of both genders were immunized by subcutaneous (sc) injection with 5 × 10^6^ plaque-forming units (PFU) of SV40 on days -14 and -7 before intra-peritoneal (ip) challenge with 10^6^ mKSA tumor cells. Growth of tumors and survival of mice were monitored up to day 100 post challenge.

Remarkably, despite immunization with SV40 all of the T1 (6 of 6; mean survival time 18 days), and all of the NP8 mice (11 of 11, mean survival time 20 days) succumbed to mKSA tumors as rapidly as naïve wtBALB/c mice (Table I).

Murine cells are not permissive for infection with SV40, allowing only abortive infection during which relatively low amounts of T-Ag are expressed. Furthermore, this abortive infection should induce only a weak inflammatory response [8, 14]. Thus, the lack of protection of T1 and NP8 mice against mKSA tumor cells could be due to the poor immunogenicity of SV40 in these mice. To test this possibility, in a repeat experiment a vaccinia virus (VV) recombinant encoding SV40 T-Ag (VV-941T) was used as a potent replicative immunogen. As expected, wt mice immunized with VV-941T were similarly protected (4/5 mice) against challenge inoculation of mKSA tumor cells (Fig. 2B). In contrast, similar to immunization with SV40, immunization with VV-941T did not protect NP8 mice. Four of five immunized mice succumbed to mKSA cells almost as rapidly as non-immunized mice (Fig. 2B). The lack of protection shows that even immunization with highly immunogenic VV-941T cannot induce T-Ag specific protective immunity in NP8 mice, indicating that transgenic expression of weakly immunogenic T-Ag interferes with induction of T-Ag specific CTLs.

**Figure 3:**
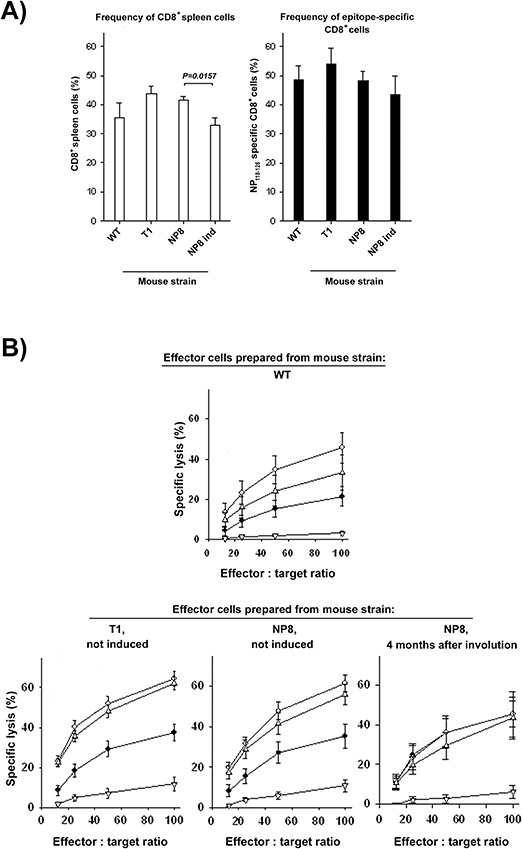
Frequency and functionality of CD8^+^ T cells in T1 and NP8 mice after acute systemic infection with LCMV Groups of wt, non-induced (naïve) T1 and NP8, or induced NP8 mice (MIN) were infected intravenously (iv) with 10^5^ PFU of LCMV. On day 8 after infection splenic single cell suspensions were prepared. **A.** The frequency of CD8^+^ (left) and of NP_118–126_-specific CD8^+^ spleen cells (right) was determined by flow cytometry using CD8-specific mAb and H-2 Ld pentamers loaded with the NP_118–126_ epitope. **B.** LCMV-specific cytotoxic activity of crude spleen cell suspensions was determined in 4 h chromium-release assays. Syngeneic BALB/c-SV fibroblasts were either infected 48 h prior to the assay with LCMV at multiplicity of infection 0.01 (♦) or loaded with 10^−6^ (◊), 10^−8^ (Δ), or 10^−10^M (∇) of the synthetic peptide epitope NP_118–126_, respectively. Shown are cumulative data from three independent experiments (numbers of mice per genotype: wt *n* = 8, T1 *n* = 7, NP8 *n* = 6, induced NP8 *n* = 3).

In contrast to the weakly immunogenic H-2^d^-restricted epitopes of T-Ag, the LCMV-derived H-2^d^-restricted, immune-dominant epitope NP_118–126_ is highly immunogenic [13]. The responsiveness of NP8 mice against this epitope, endogenously expressed within the transgenic T-Ag_NP_, was determined in mice acutely infected with LCMV (Fig. 3). The effect of transgene expression was assessed in induced NP8 mice containing intraepithelial neoplasia (MIN) and expressing large amounts of T-Ag, while effects of any spontaneous transgene expression were assessed in virgin NP8 mice. As positive controls, wt and T1 mice, both devoid of endogenous expression of the NP_118–126_ epitope, were used.

By day 8 post infection, i.e. at the peak of the LCMV-specific immune response [15], the typical expansion of total CD8^+^ spleen cells was observed in spleens of wt, T1, non-induced NP8, as well as of MIN containing NP8 mice to similar degrees (Fig. 3A, left). Moreover, among the total CD8^+^ spleen cells the frequencies of NP_118–126_-specific CD8^+^ cells were similarly elevated in wt, T1, non-induced NP8, or lactation-induced NP8 mice (Fig. 3A, right).

The LCMV-specific effector function of these splenic cells was determined in cytotoxicity assays against BALB/c fibroblast either infected with LCMV or loaded with graded concentrations of synthetic peptides representing the epitope NP_118–126_ (Fig. 3B). Furthermore, crude spleen cell populations of T1, non-induced NP8, or NP8 mice 4 months after involution (i.e. NP8 mice containing MIN) exerted similar cytotoxic activity against LCMV-infected SV40 transformed BALB/c (BALB-SV) fibroblasts (Fig. 3B). Splenocytes from infected T1 or non-induced NP8 mice lysed BALB-SV target cells loaded with graded concentrations of the synthetic NP_118–126_ peptide to similar extents. In comparison to splenocytes from T1 or non-induced NP8 mice, splenocytes from induced NP8 mice lysed target cells loaded with high concentrations (10^−6^ and 10^−8^M) of synthetic NP_118–126_ peptide slightly less effective, but this difference was not statistically significant.

The data show that in response to systemic infection with LCMV non-induced as well as induced NP8 mice effectively generate specific CD8^+^ T cells against the highly immunogenic, immune-dominant NP_118–126_ epitope with similar frequencies and cytotoxic activities as T1 and wtBALB/c mice. In contrast, even immunization with a highly immunogenic T-Ag-expressing recombinant VV induced protective immunity against T-Ag-expressing tumors only in wtBALB/c mice, but not in T-Ag-transgenic WAP-T and WAP-T_NP_ mice. This suggests that in WAP-T_NP_ mice transgenic expression of weakly immunogenic, T-Ag-derived epitopes interferes with the induction of T-Ag specific CTLs, possibly by inducing a state of hypo- or non-responsiveness, while presentation of the highly immunogenic NP_118–126_ epitope does not induce such a state. Although the reasons for this differential responsiveness to MHC class-I-restricted epitopes encoded by the same transgene are unknown to us, the strong reactivity against the NP-epitope provided us with a tool to analyze tumor-antigen specific immune responses in WAP-T_NP_ mice.

### LCMV infection induces NP-epitope specific immune responses against T-Ag_NP_ expressing cells in NP8 mice

#### LCMV infection leads to elimination of T-Ag_NP_ expressing cells in lactating NP8 mice

**Figure 4:**
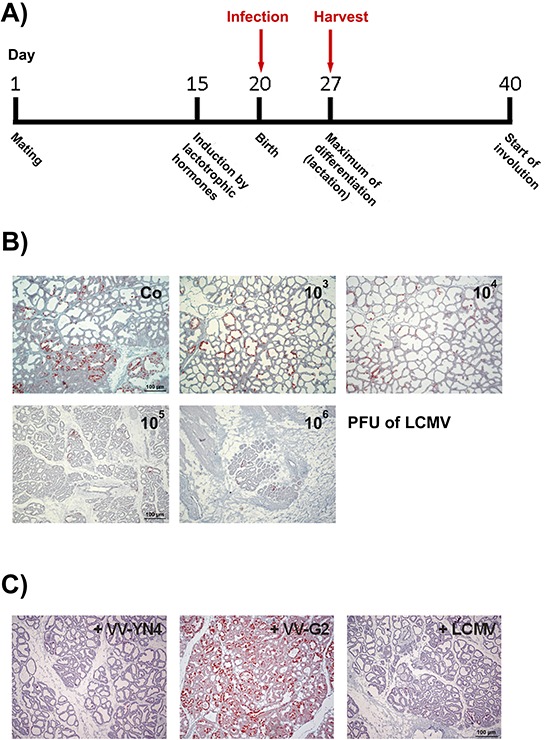
Infection of lactating NP8 mice with LCMV and VV recombinants for the analysis of a specific immune reaction against the LCMV NP_118–126_-epitope in T-Ag_NP_ **A.** Time line for harvesting the mammary glands after infection; four mice per group were infected with either different titers of LCMV or with 10^5^ PFU of LCMV or VV recombinants – the relative amounts of T-Ag expressing cells are given as percentage in brackets. **B.** Immune-histological analysis for T-Ag expression of lactating mammary glands after infection with 10^3^ [40–60%], 10^4^ [10–20%], 10^5^ [2–6%], or 10^6^ PFU of LCMV [2–6%]; the preparation shown in the left upper corner presents an uninfected control tissue (Co) [100%]. **C.** Infection of transgenic mice with 10^5^ PFU of VV recombinants containing either the NP (VV-YN4, left) [2–4%] or the glycoprotein-precursor of LCMV (VV-G2, middle) [~100%]. As control, NP8 mice were infected with LCMV (right) [2–6%].

We next asked, whether the vigorous LCMV NP-specific CD8^+^ T-cell response seen after LCMV infection of NP8 mice could eliminate endogenous T-Ag_NP_ expressing mammary epithelial cells. As the immune response against tumor cells often is compromised, we, for initial experiments, used NP8 mice in which expression of the chimeric *T-Ag/NP* transgene was induced by lactation, i.e. at a time where T-Ag_NP_ expressing cells are still normal in function. Maximal expression of T-Ag in mammary gland cells is observed between days 5–10 postpartum (pp) (Fig. 4A). After acute infection with LCMV, mature, antiviral CD8^+^ effector cells can be detected between days 6–12 post infection (pi). Accordingly, we infected maternal NP8 mice shortly after delivery and examined their mammary glands for T-Ag_NP_ expression on day 7 pp (Fig. 4B). Since the infectious dose of LCMV affects the strength of the ensuing immune response [11], we infected different groups of mice with ten-fold escalating doses of LCMV ranging from 10^3^ to 10^6^ PFU.

In mice infected with either 10^5^ or 10^6^ PFU of LCMV, virtually no T-Ag_NP_ expressing cells were detectable any more. On the other side, inflammatory reactions, as indicated in Fig. 4B by the altered histology of the mammary glands of mice infected with 10^5^ or 10^6^ PFU of LCMV, could be detected, reflecting a wide-spread LCMV infection [16]. To exclude that elimination of T-Ag_NP_ expressing cells was due to some unspecific effects of inflammation during viral infection rather than to a specific immune response against the immune-dominant LCMV epitope NP_118–126_, additional groups of mice were infected with VV recombinants encoding either the LCMV glycoprotein precursor (VV-G2) or the LCMV nucleoprotein (VV-YN4) (Fig. 4C) [13]. Similar to infection with LCMV (Fig. 4C, right), after infection with VV-YN4 only few remaining T-Ag_NP_ expressing cells were detectable (Fig. 4C, left). In contrast, infection with VV-G2, encoding the LCMV glycoprotein precursor, did not lead to a reduction of T-Ag_NP_ positive cells (Fig. 4C, middle). The specificity for NP-epitope specific elimination of T-Ag_NP_ expressing cells was further confirmed by additionally infecting the mice with VV virus recombinant VV-941T encoding the SV40 T-Ag (Supplementary Fig. 1). As expected from the data shown in Figs. 2 and 3, VV-941T was not able to induce elimination of T-Ag_NP_ expressing cells.

The data show that infection with either LCMV or VV-YN4, both expressing the LCMV nucleoprotein, specifically leads to NP-epitope specific elimination of T-Ag_NP_ expressing cells due to induction of an NP-epitope specific T-cell response that eliminates T-Ag_NP_ expressing cells.

#### Adoptive transfer of LCMV NP-specific splenocytes into lactating NP8 mice leads to selectivse elimination of T-Ag_NP_ expressing mammary epithelial cells

**Figure 5:**
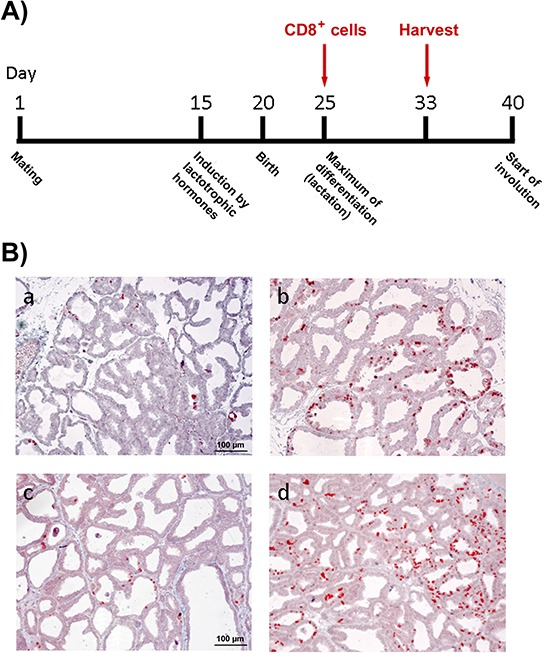
Adoptive transfer of immune cells from infected and uninfected BALB/c into NP8 mice **A.** Scheme depicting the time point of splenocyte transfer from five individually infected BALB/c mice into the corresponding number of transgenic mice and harvest of their mammary glands; the calculated relative amounts of T-Ag positive cells are shown as percentage in brackets. **B.** NP8 mice received either the spleen cells of BALB/c mice infected with the VV recombinants containing the NP of LCMV (VV-YN4, c) [2–4%] or the T-Ag of SV40 (VV-941T, d) [~100%]; mammary glands of NP8 mice, which received spleen cells from LCMV-infected BALB/c mice (LCMV, a) [2–6%] or none (Co, b) [100%] served as positive and negative controls.

To provide stringent evidence that elimination of T-Ag_NP_ expressing cells is due to the activity of immune cells rather than to some unspecific inflammatory response of the host, we performed adoptive transfers of fully active effector cells from acutely LCMV-infected wtBALB/c into lactating recipient NP8 mice. Spleen cells from donor mice infected 7 days earlier were adoptively transferred into NP8 mice at day 5 pp (Fig. 5A). Eight days after transfer, mammary glands of recipients of spleen cells from LCMV infected donors (Fig. 5B, panel a) showed a strong reduction in the number of T-Ag_NP_ expressing cells as compared to non-transferred controls (Fig. 5B, panel b). Specificity of this effect for the LCMV NP as immunogen was proven by a similarly quantitative reduction of T-Ag_NP_-expressing cells after transfer of spleen cells from donor mice infected with VV-NY4 (Fig. 5B, panel c), while transfer of spleen cells from donors infected with VV-941T did not conspicuously reduce the density of Tag-expressing cells (Fig. 5B, panel d).

Altogether the data show that specific immune responses against the LCMV NP-derived, immune-dominant epitope NP_118–126_ lead to a strong reduction of T-Ag_NP_ expressing mammary gland cells in lactating NP8 mice.

#### LCMV infection leads to elimination of T-Ag_NP_ expressing tumor cells in NP8 mice

**Figure 6:**
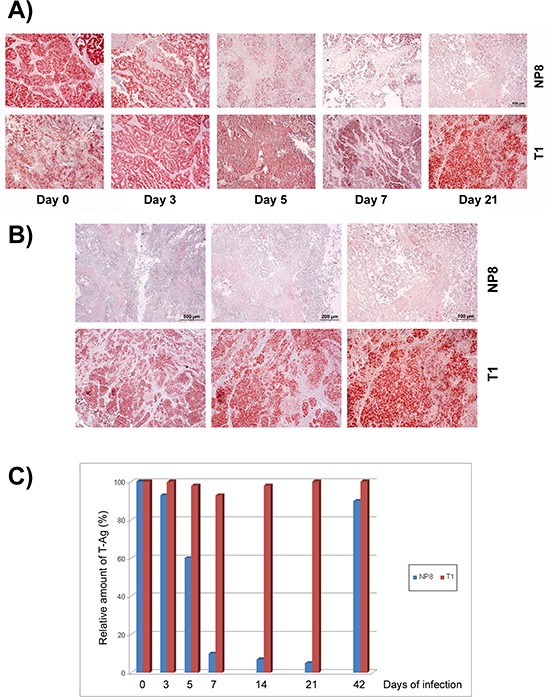
Specific elimination of T-Ag_NP_ expressing mammary tumor cells after LCMV infection **A.** NP8 mice (upper panels) or T1 mice (lower panels) bearing mammary tumors (diameter about 1.0 cm) were infected with 10^5^ PFU of LCMV and tumors of mammary glands were prepared and stained for T-Ag at days 0, 3, 5, 7, 14 (not shown), 21, and 42 (not shown). **B.** Comparison of T-Ag expression (different magnifications) in tumors of NP8 (upper panels) and T1 mice (lower panels) 21 days after infection with LCMV. **C.** Demonstration of the transient elimination of tumor cells in NP8 mice (blue columns) and T1 mice (red columns) after virus infection by the loss of tumor-derived T-Ag (visual calculation of different fields within the stained samples).

**Figure 7:**
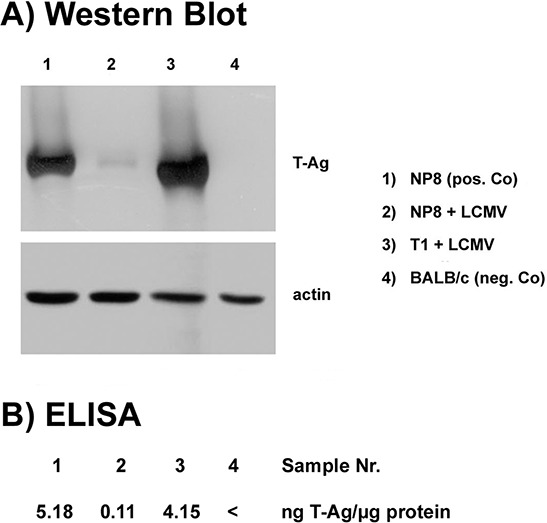
Quantitative comparison of T-Ag expression in tumors after infection with LCMV Amounts of T-Ag were measured after infection with 10^5^ PFU NP8 (2) and T1 (3) mice in tumor lysates at day 7 pi; uninfected NP8 tumor (1), and BALB/c mice (4) served as positive and negative controls, respectively. Reduction of T-Ag levels in tumor lysates after LCMV infections of NP8 and T1 mice was examined in parallel by Western blotting **A.** or by ELISA **B.**

In the previous experiments the specific immune response was initiated during development and expansion of pre-cancerous mammary epithelial cells. Next we assessed, whether LCMV-infection could also induce a specific immune response in T-Ag_NP_ expressing tumors of NP8 mice. T-Ag expressing tumors in T1 mice served as control. T1 and NP8 tumor mice (tumor diameter about 1 cm), respectively, were systemically infected with LCMV and tumors analyzed at days 0, 3, 5, 7, and 21 pi (Fig. 6). At day 0 of infection, in tumors of both, NP8 and T1 mice, large areas of densely packed T-Ag expressing cells were observed. In T1 control mice, the pattern of T-Ag positive cells within the tumors remained almost unaffected up to day 21 pi. The somewhat disorganized histology of the mammary gland in LCMV infected T1 mice at day 7 most likely reflects inflammatory reactions induced by LCMV infection. In contrast, as early as day 3 pi, the density of T-Ag_NP_ positive cells was already slightly decreased in tumors of NP8 mice, and from day 5 to 21 pi only individual T-Ag_NP_ positive cells, scattered within the tumor mass, were detected (Figs. 6A, 6B). For a better evaluation, tissue sections of T1 and NP8 tumor mice were compared at day 21 pi with different magnifications, where the right panels represent the magnification applied in Fig. 6A (and in all other Figs), whereas, for a better overview, the panels in the middle show a 2.5-fold and the panels on the left side a 5-fold lower magnification (Fig. 6B). Semi-quantitative evaluation of T-Ag positive cells in Fig. 6A (i.e. relative numbers of T-Ag expressing cells per section, for details see legend to Figs 4 and 5) graphically shows a rapid decline after LCMV infection in tumors of NP8 mice, while the fraction of T-Ag positive cells in tumors of T1 mice stayed relatively constant (Fig. 6C). Elimination of T-Ag_NP_ expressing cells in NP8 tumors after systemic infection with LCMV was additionally quantified in a subsequent experiment at day 7 pi by Western blot (Fig. 7A) and ELISA of tumor lysates (Fig. 7B) for T-Ag and revealed a strong reduction, while T-Ag levels in T1 tumors remained unaffected (Fig. 7B).

These data show that an effective LCMV NP-epitope specific T-cell response can also be induced in tumor-bearing NP8 mice. However, reduction of T-Ag_NP_ expressing tumor cells was transient. Despite the initially almost quantitative elimination of tumor cells in NP8 mice after LCMV infection, tumors reappeared about 6 weeks after treatment (Fig. 6C, 42 days). Transient reduction of T-Ag_NP_ expressing cells could be achieved repeatedly by consecutive LCMV infections, but tumors always grew back (data not shown).

### Analysis of spontaneous NP-epitope specific CD8^+^ T-cell responses in WAP-T_NP_ mice

T-Ag in T1 and T-Ag_NP_ in NP8 mice are encoded by their respective WAP-driven transgenes and thus have to be considered “self”-proteins. Usually, mammals establish tolerance or anergy against self-proteins during their development. In line, T1 and NP8 mice both lacked detectable T-Ag specific immunity. In contrast, we observed a strong LCMV NP-epitope specific immunity in NP8 mice. This may be suggestive for differential tolerance versus immunogenicity of specific antigenic determinants. We thus considered the possibility that WAP-T_NP_ mice *a priori* were not tolerant against the NP-epitope in T-Ag_NP_. Therefore, we addressed the question, whether in WAP- T_NP_ mice NP-epitope specific immune responses are detectable without exogenous immunization.

#### Treatment of WAP-T_NP_ NP6 mice with anti-CD8 antibodies or sub-lethal irradiation leads to an increase of T-Ag_NP_ expressing mammary epithelial cells after involution

**Figure 8:**
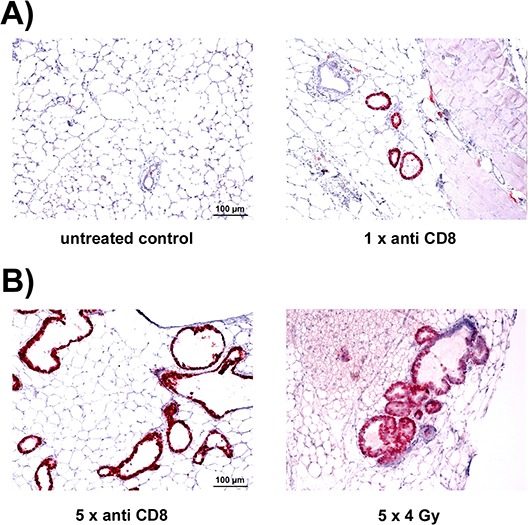
Immune depletion enhances the amount of T-Ag expressing mammary cells in NP6 mice after involution **A.** Increase after a single treatment with 400 μg of anti CD8 antibodies at day 1 pp and analysis at day 20 pw (right) in comparison to an untreated NP6 mouse (left). **B.** Increase of T-Ag expressing mammary epithelial cells in involuted NP6 mice by repeated treatments with anti CD8 antibodies (left) or sub-lethal irradiation (right) analyzed 20 days after the last handling.

In WAP-T_NP_ mice, the first appearance of T-Ag_NP_ positive cells is late in pregnancy and then during lactation in mammary glands of induced mice. If WAP-T_NP_ mice were not tolerant against the NP-epitope, NP-specific CD8^+^ T-cells should be generated latest during lactation phase, and in consequence should lead to an elimination of T-Ag_NP_ expressing cells later on, i.e. in involuted glands. If this were the case, elimination of CD8^+^ T-cells in WAP-T_NP_ mice after birth of offspring should enhance the frequency of T-Ag_NP_ positive cells in involuted glands. To test this hypothesis we used NP6 mice (for a detailed description of NP6 mice see Materials and methods), as T-Ag_NP_ expressing cells are scarce (i.e. in most cases not detectable at all) in involuted mammary glands of NP6 mice (Fig. 8A, left). Thus an increase in T-Ag_NP_ positive cells after immune-suppressive treatment would be much easier to detect than in NP8 mice in which the majority of ducts contain T-Ag_NP_ positive cells. We treated NP6 mice at day 1 pp with anti-CD8 antibodies and checked at day 20 post weaning (pw) for changes in the amount of T-Ag_NP_ expressing cells in involuted mammary glands. Fig. 8A, right panel, shows that already a single dose of anti-CD8 antibodies significantly increased the number of T-Ag_NP_ positive cells. Continuous treatment by repeated injections of anti-CD8 antibodies, beginning at birth and then every 20 days, led to an even stronger increase in T-Ag_NP_ positive ducts in involuted glands (Fig. 8B, left), when observed 100 days pp. Sub-lethal irradiations of NP6 mice, i.e. a more generalized immune-suppression, performed in parallel at the same time intervals led to a similar increase in T-Ag_NP_ positive cells (Fig. 8, right). The data suggest that WAP-T_NP_ mice mount a spontaneous immune response against the NP-epitope and thus are not tolerant against the NP-epitope in T-Ag_NP_.

#### NP8 tumor mice contain CD8^+^ T-cells against the NP- epitope

**Figure 9:**
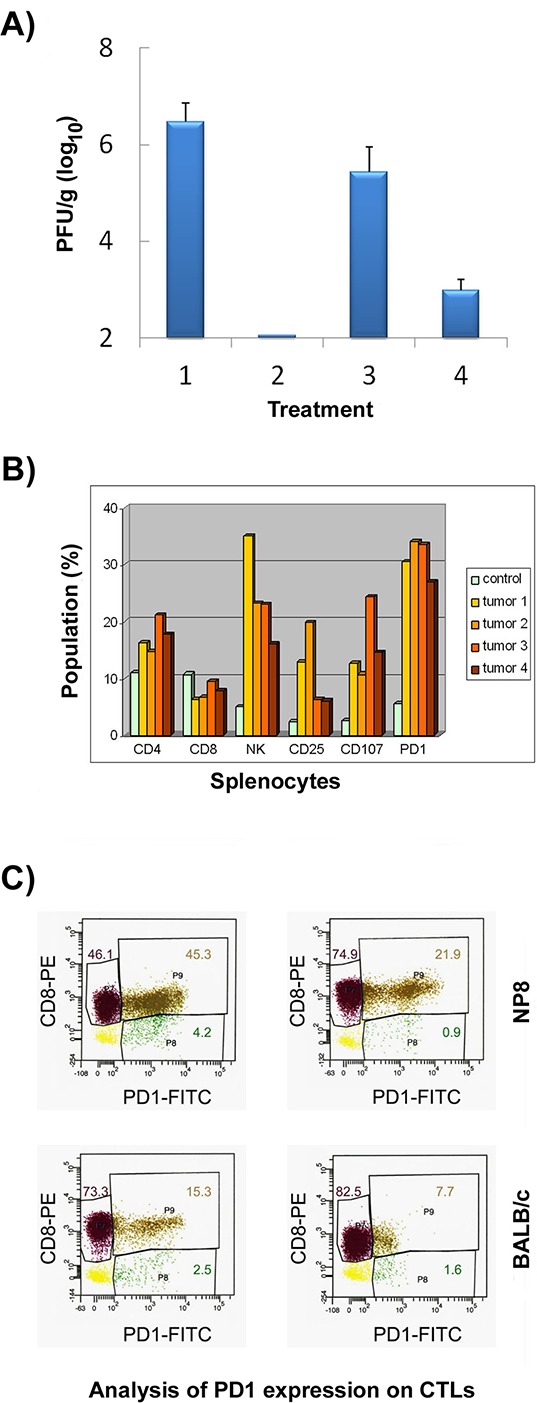
Detection of NP-epitope specific CTLs in NP8 tumor mice **A.** Adoptive transfer of immune cells into LCMV-infected BALB/c mice. CTLs were obtained from BALB/c mice infected with LCMV (2, positive control), from NP8 tumor mice (3), from NP8 tumor mice and applied together with anti-PD1 antibodies (4); untreated BALB/c mice were introduced as negative controls (1); the means and standard errors of 5 mice are shown. **B.** Examination by FACS of splenocytes from wtBALB/c and NP8 tumor mice. Splenocytes from one BALB/c (control) and four NP8 tumor mice (tumors 1–4) were isolated and studied by FACS. **C.** Splenocytes from two NP8 tumor mice and two BALB/c control mice were sorted with anti-CD8 antibodies. Then these pre-selected cells were stained with anti-PD1 antibodies for a final analysis via FACS. The results revealed an increase of CD8^+^PD1^+^ cells (brown) of 45.3 and 21.9% versus CD8^+^PD1^−^ cells (violet) of 46.1 and 74.9% in NP8 tumor mice in comparison to CD8^+^PD1^+^ cells (brown) of 15.3 and 7.7% versus CD8^+^PD1^−^ cells (violet) of 73.3 and 82.5% in wtBALB/c mice; the minor fractions presented here were CD8^−^PD1^+^ cells (green) between 0.9 and 4.2% and CD8^−^PD1^−^ cells or cellular debris (yellow) between 2.3 and 8.9% in all mice, respectively.

We next asked, whether we can detect NP-epitope specific CD8^+^ T-cells also in NP8 tumor mice. Adoptive transfer of splenocytes from NP8 tumor mice into LCMV infected BALB/c mice and the possible reduction in virus titer 7 days later served as read-out system. As positive control we used splenocytes from LCMV infected BALB/c mice, harvested 7 days after infection with LCMV (Fig. 9A). While the titer of LCMV in untreated control mice remained at a mean of 3 × 10^6^ PFU/g of tissue (Fig. 9A, lane 1), transfer of splenocytes from LCMV infected BALB/c mice eliminated LCMV from the spleen of infected mice below the level of detectability (Fig. 9A, lane 2). Transfer of splenocytes from NP8 tumor mice reproducibly reduced virus load by about one log_10_ (Fig 9A, lane 3), i.e. by about 90%, indicative for a significant, though comparatively weak anti-NP-epitope specific CTL activity in these mice. We conclude that NP8 tumor mice contain CD8^+^ T-cells against the NP-epitope which elicit a weak, but measurable anti-NP-epitope specific CTL activity.

#### Splenocytes in NP8 tumor mice contain a large population of PD1 expressing cells

To get more information on the immune status of NP8 tumor mice, we compared the composition of splenocytes from wtBALB/c and NP8 tumor mice. Fig. 9B shows an enhanced frequency of CD4^+^ T cells in NP8 tumor compared to wtBALB/c mice, while the fraction of CD8^+^ T cells is rather similar in wtBALB/c and in NP8 tumor mice. Prominent increases in NP8 tumor mice were found in the fractions of CD25^+^ cells, which could explain the enhanced frequency of CD4^+^ T cells in NP8 tumor compared to wtBALB/c mice, as CD25^+^ cells also include CD4^+^ regulatory T-cells. Furthermore, an enhancement was observed in NK cells and in CD107^+^ positive cells, indicative for the presence of activated NK and CD8^+^ T-cells. The most striking observation, however, was the strong increase in the frequency of cells expressing the programmed death-1 (PD1) molecule. PD1 is part of a pathway negatively regulating immune responses and can be expressed on activated T and B cells, on mesenchymal stem cells, but is highly expressed on exhausted CD8^+^ T cells [17], thereby impeding their activity. To determine the fraction of PD1^+^ positive cells within the CD8^+^ T-cell compartment, we first sorted splenocytes for CD8^+^ T-cells. Sorted CD8^+^ T-cells then were stained with anti-PD1 antibodies and again analyzed by FACS (Fig. 9C), which revealed a strong increase of PD1^+^ cells within the pre-selected CD8^+^ compartment in NP8 tumor mice (23% and 50%) in comparison to wtBALB/c mice (9% and 17%).

#### Anti-PD1 treatment functionally reactivates exhausted NP-epitope specific T-cells in NP8 tumor mice

To test, whether the activity of NP-epitope specific CD8^+^ T cells in NP8 tumor mice indeed is impaired by expression of PD1, NP8 tumor mice were treated once with (50 μg) anti-PD1 antibodies. At day 7 after treatment, splenocytes were harvested and used for adoptive transfer into LCMV infected BALB/c mice. Fig. 9A, lane 4 demonstrates a significant reduction of the virus titer in these mice. As in BALB/c mice NP-epitope specific CTLs are the only immune cells responsible for LCMV elimination, we conclude that anti-PD1 treatment of splenocytes from NP8 tumor mice leads to a re-activation of CD8^+^ T-cells, whose activity in tumor mice is compromised by expression of PD1.

The data demonstrate that NP8 tumor mice contain weakly reactive NP-epitope specific CD8^+^ T-cells; the majority of those, however, is exhausted by expression of PD1, but can be at least partially reactivated by treatment with anti-PD1 antibodies.

## DISCUSSION

Immune-therapy of cancers holds great promise, but up to now tumor-induced mechanisms that lead to immune-evasion pose major barriers to its successful application [18]. Analysis of the immune status of a given tumor entity and identification of the obstructed immune checkpoints thus are crucial issues for the development of immune-therapeutic anti-cancer strategies. Due to the limited possibilities for analyzing the respective parameters in humans, suitable animal models should be of great value. While a large number of studies has shown that it is possible to protect mice against tumor transplants by pre-immunization with the respective tumor antigens (protective immunization), very few studies so far have examined, whether and how the growth of naturally arising tumors can be controlled by inducing a tumor antigen specific immune response (curative immunization).

In this study we focused on epitope-specific CD8^+^ T-cell responses against WAP-T mammary epithelial cells expressing a chimeric SV40 T-Ag, carrying the H-2^d^ restricted NP-epitope of LCMV (T-Ag_NP_). While the CTL-immune-response of BALB/c mice against T-Ag specific H-2^d^ restricted T-cell epitopes is very weak, though measurable [10], the LCMV NP-epitope is the strong, singular immune-dominant CD8^+^ T-cell epitope in BALB/c mice [11, 13, 19]. Our rationale was that this immune-dominant epitope, inserted into a transformation-irrelevant region of SV40 T-Ag, might allow the induction of a strong CD8^+^ T-cell response in WAP-T_NP_ mice after immunization with LCMV. Taking advantage of immune-dominant epitopes in analyzing epitope-specific CTL-responses against endogenously arising tumors is not a new approach. Speiser et al. [20] and Schell et al. [21] already demonstrated that it is possible to override epitope-specific tolerance and mount a CTL-specific anti-tumor immune-response by immunization with a strong immunogen expressing the respective CTL-epitope.

To analyze NP-epitope specific T-cell responses in NP8 mice we used two different approaches, infection of the mice with LCMV or NP-epitope expressing VV recombinants, and transfer of splenocytes from LCMV infected wtBALB/c mice. Both approaches unequivocally demonstrated that NP-epitope specific CD8^+^ T-cells specifically eliminate T-Ag_NP_ expressing cells in NP8 mice both prior to as well as after tumor development. Elimination of tumor cells after LCMV infection of NP8 tumor mice was almost complete, as judged by immune-histochemistry and by measuring the T-Ag_NP_ content in tumor lysates. However, this elimination was only transient, as starting with day 21 after LCMV treatment tumors recurred. Even repeated immunizations with LCMV did not succeed to achieve long term immunity. This finding was similar as in RIP(Tag2 × GP) mice [20], where immunization with LCMV also led to strong tumor reduction, but not to complete and long lasting elimination.

The stringent NP-epitope specificity of CTLs observed in NP8 mice after infection with LCMV or after adoptive transfer of splenocytes from LCMV infected BALB/c mice is based on several arguments: (1) the NP-epitope contained within the chimeric T-Ag_NP_ protein is the only LCMV-specific epitope present in NP8 mice; (2) NP8 mice showed a virus dose-dependent reduction of the T-Ag load in the tissues of mammary glands (Fig. 4A); (3) specific elimination of T-Ag expressing cells was observed only by a vaccinia virus recombinant expressing the NP molecule of LCMV but not with recombinants expressing either the glycoprotein-precursor of LCMV or the T-Ag of SV40 (Fig. 4B and Supplementary Fig. 1); (4) reduction of T-Ag positive cells could be achieved by adoptive transfer of immune cells only when lymphocytes were used from BALB/c mice infected with LCMV or VV-YN4 (Fig. 5); (5) specific elimination of T-Ag expressing tumor cells was observed by LCMV infection of individual NP8 tumor mice but not T1 mice (Fig. 6).

The novel finding in our system was that induced as well as tumor bearing WAP-T_NP_ mice showed an endogenous NP-epitope specific CTL-response, and thus that these mice were not tolerant against this epitope. This conclusion had been already suggested by our initial experiments demonstrating that NP8 mice were able to mount a very strong, NP-epitope specific immune response after LCMV infection (Table I and Fig. 2). This finding was somewhat surprising, as the chimeric T-Ag_NP_ is expressed from a transgene and thus should be treated immunologically as a “self”-protein. We do not have a conclusive explanation, especially since in the studies by Speiser et al. [20] and Schell et al. [21] cited above non-immunized mice were tolerant against the analyzed immune-dominant CTL-epitopes. However, an important difference between their animal models and ours is that in their mice the transgenes are continuously expressed starting with organ development, while T-Ag_NP_ in WAP-T_NP_ mice is first expressed very late in pregnancy and then strongly during lactation and later on in tumors. In this respect our model more closely mimics naturally occurring tumors in humans, where tumor neo-antigens are also presented only in cancer.

The NP-epitope specific immune status of WAP-T_NP_ mice may bear similarity to the antigen specific immune status in chronic infection [22, 23], where the CD8^+^ T-cell response is down-regulated, but not completely abolished by surface receptors of negative regulatory pathways, e.g. PD1. In line with this idea we found that WAP-T_NP_ mice are able to mount a spontaneous CD8^+^ T-cell response against the NP-epitope in T-Ag_NP_. Treatment of NP6 mice after birth of offspring with anti-CD8 antibodies or sub-lethal irradiation provided a first hint for the existence of reactive, NP-specific CD8^+^ T-cells, as such treatment led to a strong increase in T-Ag_NP_ positive cells after involution. Furthermore, NP8 tumor mice contained reactive NP-epitope-specific CD8^+^ T-cells, as splenocytes from such mice after adoptive transfer weakly, though measurably reduced the virus load in LCMV infected wtBALB/c mice. This inadequate endogenous immune response suggested that NP-specific CD8^+^ T-cells in NP8 tumor mice were in a weakly reactive state, thereby explaining why they were not able to prevent tumor outgrowth. In support, FACS analyses of splenocytes from NP8 tumor mice revealed that a large fraction of the immune cells, specifically of CD8^+^ T-cells, expressed PD1, a commonly accepted marker for T-cell exhaustion [17, 24]. However, as recently demonstrated by Utzschneider et al. [23] in chronic LCMV infection, PD1 mediated exhaustion of CD8^+^ T-cells is not absolute and could mark a specific state of CD8^+^ T-cell differentiation in a given immunological setting (e.g. in chronic infection) rather than an irreversible pathway of CD8^+^ T-cell elimination. This view is supported by our findings that already adoptive transfer of untreated splenocytes from WAP-T_NP_ tumor mice into LCMV infected wtBALB/c mice led to a slight reduction in LCMV titer in BALB/c mice (Fig. 9). Adoptive transfer of anti-PD1 treated splenocytes from WAP-T_NP_ tumor mice into LCMV infected BALB/c mice then led to a significant re-activation of NP-epitope specific cytotoxic CD8^+^ T-cells, underscoring the role of PD1 expression in the control of CTL activity.

We interpret our data as to indicate that transgene expressing NP8 mice contain NP-epitope specific CTLs, which, however, are only partially reactive due to expression of PD1, and thus are not sufficient to control tumor growth. In analogy, we consider it likely that the only transient elimination of NP8 tumor cells in NP8 tumor mice after LCMV infection also reflects PD1 mediated exhaustion of NP-epitope specific CD8^+^ T-cells generated during infection, i.e. the restoration of the compromised immune-status in tumor mice by repeated antigen stimulation. Very recently, anti-PD1 treatment of NP8 tumor mice has evolved as a promising approach for re-activation of the spontaneous immune response against tumors [25]. However, such a treatment must be coupled with measures that effectively prevent the re-establishment of the PD1-mediated compromised immune-status.

The WAP-T_NP_ model described in this study to our best knowledge is unique insofar that these mice are not tolerant to a transgene-encoded tumor antigen. It thus may serve as a model for human tumors, as it is well known that human tumors can mount specific CTL-responses against their tumor antigens. The probably best known tumor entity in this respect are melanomas, and boosting their cellular immune response by various approaches has been used in immune therapy, though previously with limited success [26], if applied without measures restoring compromised immune checkpoints [25]. It is most likely that many human tumors develop a spontaneous cellular immune response against tumor antigens, which prospectively could be used for immune therapy. Thus the WAP-T_NP_ model might be helpful in deciphering the parameters that will finally allow successful curative immunization strategies.

## MATERIALS AND METHODS

### Mice

For our analyses, we selected the WAP-T mouse line T1 as an NP-epitope negative control, and two WAP- T_NP_ mouse lines, WAP-T-NP6 (NP6) and WAP-T-NP8 (NP8), respectively [5]. If not stated otherwise, generally five mice per group were used in each experiment. NP6 and NP8 mice differ in their characteristics of tumor formation: NP8 mice are very similar to T1 mice [2], strongly expressing the SV40 T-Ag in about 50% (NP8) to about 90% (T1) of epithelial cells of lactating mammary glands (Fig. 1B). The majority of T-Ag positive cells are eliminated during involution, but proliferation of T-Ag expressing progenitor cells that survived involution soon leads to the appearance of T-Ag positive ducts. Consequently, virtually all terminal end buds in T1 and NP8 mice develop intraepithelial neoplasia (MIN) within 90–120 days post weaning (pw) [6, 27]. Within the same time frame (6–8 months) T1 and NP8 mice develop between 2 to maximally 6 (average 3) invasive mammary carcinomas. In contrast, in induced NP6 mice only very few T-Ag expressing cells (about 2%) are found in ducts of lactating mammary glands (Fig. 1B). Correspondingly, only very few T-Ag_NP_ positive cells can be detected in mammary glands of NP6 mice after involution, and only a few MIN, but no outgrowth to invasive carcinomas are observed. However, after a two-fold induction 90% of NP6 mice also develop invasive carcinomas, which arise significantly later than in T1 or NP8 mice. No metastases were found in two-fold induced NP6 tumor mice (unpublished data).

BALB/c and transgenic mice were held under specific pathogen-free conditions, and the generation of WAP-T or WAP-T_NP_ mice was described in detail elsewhere [5]. The WAP promoter is hormonally and developmentally regulated by lactotrophic hormones (e.g. estrogen, prolactin, hydrocortison, insulin) [28]. Thus expression of the transgene can be induced by mating and is directed to epithelial cells of ducts of the differentiating and lactating mammary glands. Before adoptive transfer of immune cells, acceptor mice were γ-irradiated sub-lethally with a radiation dose of 4 Gray by the Cs-137 source (2,500–3,000 Curie) of a LISA I apparatus (Conservatome). Similarly, in some cases NP6 mice were dealt with sub-lethal irradiations or were alternatively treated ip with 400 μg of monoclonal antibodies (mAbs) against anti-CD8 [29, 30]. For the specific removal of exhausted T cells mice were treated with 50 μg anti-PD1 mAbs (eBioscience).

### Virus and cells

The plaque-purified WE strain of LCMV [31], SV40 [32], and VV as well as VV recombinants [13, 21] were propagated and titrated in mouse NCTC clone 929 L cells using minimal essential medium supplemented with nonessential amino acids and 5% heated calf serum at 37°C [33]; the virus was quantitatively expressed as numbers of plaque-forming units (PFU) [34]. Mice were, if not stated otherwise, infected with 10^5^ PFU of virus intravenously (iv), ip, sc, or into the foot-pad according to described procedures [30]. Donor BALB/c mice were infected with 10^2^ PFU of virus before transfer of splenocytes into acceptor mice.

### Immune histochemistry

Histopathology and analysis of transgene expression were essentially as already described [5]. In brief, mouse mammary tissue specimens were fixed with 4% formaldehyde containing 1% acetic acid and embedded in paraffin. Deparaffinated sections were stained with hematoxylin and eosin. Immunostaining of SV40 large T-antigen was performed on paraffin sections using a triple-step immunoenzymatic method. Deparaffinated sections were reacted before antibody incubation with a commercial ‘target unmasking fluid’ (Dianova) in a microwave oven. Subsequently, sections were incubated overnight at 48°C with a 1 : 10,000 dilution of the polyclonal rabbit antiserum R15 against T-Ag [35]. Specifically bound primary antibody was detected using biotinylated anti-rabbit IgG and phosphatase-conjugated streptavidin from a commercial kit (Super Sensitive Detection System, Biogenex). Phosphatase enzyme activity was revealed with naphthol AS-BI phosphate in combination with hexazotized new fuchsine (Merck). Naïve rabbit serum served as control. Sections were slightly counterstained with hemalum. All photographs were taken by the Zeiss Axioplan2 imaging microscopic equipment with the camera ProgRes C12plus of Jenoptic using the Software ProgRes CapturePro 2.9.0.1.

### FACS

Analysis of splenocytes were performed usually with 5 × 10^5^ cells per staining. Cells were washed and resuspended in 100 μl PBS buffer; thereafter 1 μl of FITC- or PE-stained antibodies (BD Biosciences) were added according to the manufacturer's instructions. For the examination of the relevant cellular populations the following mAbs, all obtained from BD Biosciences, were used: rat anti-mouse CD8 as well as CD4, mouse anti-mouse NK1.1, rat anti-mouse CD25, rat anti-mouse CD107a, and hamster anti-mouse PD1 and incubated in the dark for 2 h. In order to inhibit unspecific binding rat anti-mouse CD16/CD32 was included to each arrangement as Fc block. Appropriate rat anti-mouse IgG1, IgG2a, and IgG2b were introduced as isotype controls. Cells were resuspended in 500 μl FACS buffer (0.5% FCS, 100 μM EDTA in PBS) and analyzed in a FACSAria I cell sorter (Becton Dickinson) with BD FACS Diva 5.1.3 software. For analysis of the fraction of CTLs expressing the PD1 protein CD8^+^ cells were first sorted after incubation of splenocytes with anti-mouse CD8 antibodies for 2 h in the dark and then examined by FACS using the anti-mouse PD1 antibodies described above.

### Detection of LCMV-specific cytotoxic T-cells

The activity of CTLs was quantified by standard 4 h ^51^Cr-release assays according to Brunner and coworkers [36] with the modification that the incubation time for the target cells was diminished to 4 h at 37°C as already mentioned [30]. The specificity of the immune reaction within the BALB/c background was verified by the LCMV NP pentamer haplotype with peptide sequence H-2Ld / RPQASGVYM. The quantity of discharged radioactive label was measured with a γ-ray scintillation counter (Berthold, Packard) and the specific release was calculated with the formula [100 × (a-b)/(c-b)] − [100 ×(d- b)/(c-b)], where a = counts of antigen-positive target cells co-incubated with immune lymphoid cells, d = counts of non-infected target cells in the presence of immune lymphoid cells, b = counts of target cells only, and c = counts of lysed target cells.

### Protein measurement and detection of T-Ag in ELISA and western blotting

The procedures were already described in more details [37]. Briefly, the protein content was calculated using the Bio-Rad protein assay with the Bradford Reagent [38]. For the determination of the amounts of T-Ag an ELISA was carried out, where aliquots of the samples were adsorbed onto MaxiSorp Immunoplates (Nunc) for 2 h at room temperature. The detection of viral antigen was performed with the rabbit anti T-Ag antiserum R15 [35] followed by horseradish peroxidase-labeled goat anti rabbit immunoglobulins (Medac). The presence of T-Ag was also visualized after electrophoretic separation of whole cell lysates in 5 to 20% polyacrylamide gradient gels [39] and blotting onto PVDF membranes (Bio-Rad), which were incubated with anti-T-Ag antiserum R15 diluted 1:1000.

## SUPPLEMENTARY FIGURE



## Abbreviations

Co: control
Co: control
CTL: cytotoxic T lymphocyte
HE: hematoxylin and eosin
ip: intraperitoneal
iv: intravenous
LCMV: lymphocytic choriomeningitis virus
mAb: monoclonal antibody
MHC: major histocompatibility comples
MIN: mammary intraepithelial neoplasia
mutp53: mutant p53
NK: natural killer cell
NP: nucleoprotein of LCMV
NP6: WAP-T-NP6 mouse
NP8: WAP-T-NP8 mouse
PD1: programmed death-1 protein
PD-L1: ligand of PD1
PFU: plaque-forming units
pi: post infectionem
pp: postpartum
pw: post weaning
sc: subcutaneous
SV40: Simian virus 40
T1: WAP-T1 mouse
T-Ag: T-antigen of SV40
T-Ag_NP_: chimeric T-Ag/NP protein
VV: vaccinia virus
VV-941T: VV containing SV40 T-Ag
VV-G2: VV containing LCMV glycoprotein precursor
VV-YN4: VV containing LCMV nucleoprotein
WAP: whey acidic protein
wt: wild type.
