# Supplementary material for: An inducible transgenic mouse breast cancer model for the analysis of tumor antigen specific CD8+ T-cell responses
Source: Oncotarget. 2015 Oct 19;6(36):38487–503. doi: 10.18632/oncotarget.5750 (PMC4770716; doi:10.18632/oncotarget.5750)
Supplement: Supplementary file 1 [file oncotarget-06-38487-s001.pdf]

SUPPLEMENTARY FIGURE

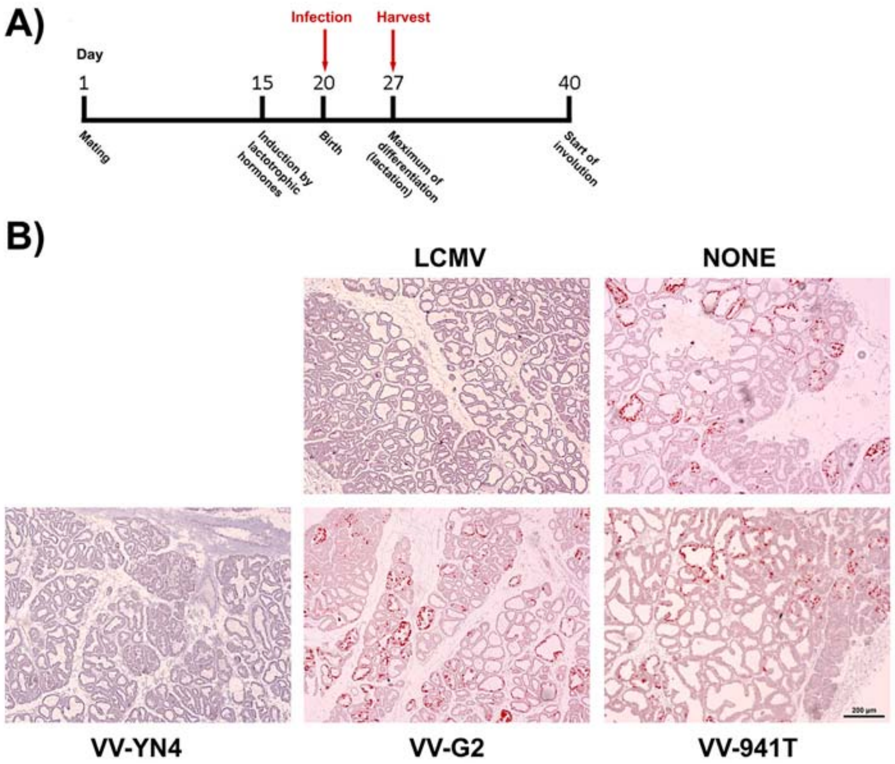

Supplementary Figure S1: Infection of lactating NP8 mice with LCMV and VV recombinants for the analysis of a specific immune reaction against the LCMV NP$_{118–126}$-epitope in T-Ag$_{NP}$. A. Time line for harvesting the mammary glands after infection; four mice per group were analyzed by immune histology after staining with anti-T-Ag antibodies; a representative experiment is shown. **B** Infection of transgenic mice with 10^5 PFU of VV recombinants containing either the glycoprotein-precursor (VV-G2), the NP of LCMV (VV-YN4) or the T-Ag of SV40 (VV-941T). As negative and positive controls, the same number of not infected (None) as well as with 10^5 PFU of LCMV infected NP8 mice (LCMV) were examined in parallel.
